# Supplementary material for: Rest/stress myocardial perfusion imaging by positron emission tomography with 18F-Flurpiridaz: A feasibility study in mice
Source: J Nucl Cardiol. 2022 Apr 28;30(1):62–73. doi: 10.1007/s12350-022-02968-9 (PMC9984310; doi:10.1007/s12350-022-02968-9)
Supplement: Supplementary file 1 — Electronic supplementary material 1 (PPTX 1033 kb) [file 12350_2022_2968_MOESM1_ESM.pptx]

## Slide 1
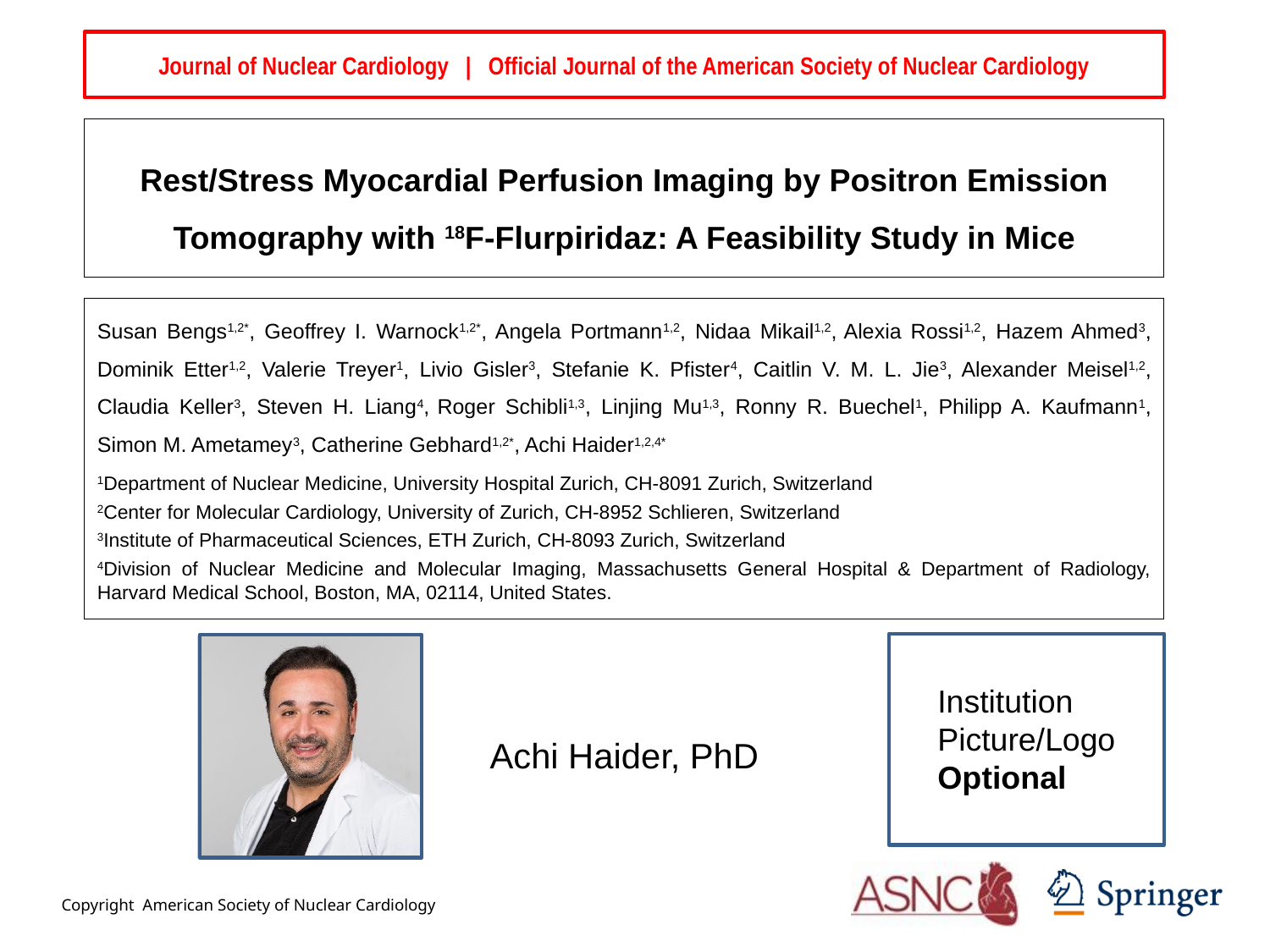

Journal of Nuclear Cardiology | Official Journal of the American Society of Nuclear Cardiology
# Rest/Stress Myocardial Perfusion Imaging by Positron Emission Tomography with 18F-Flurpiridaz: A Feasibility Study in Mice
Susan Bengs1,2*, Geoffrey I. Warnock1,2*, Angela Portmann1,2, Nidaa Mikail1,2, Alexia Rossi1,2, Hazem Ahmed3, Dominik Etter1,2, Valerie Treyer1, Livio Gisler3, Stefanie K. Pfister4, Caitlin V. M. L. Jie3, Alexander Meisel1,2, Claudia Keller3, Steven H. Liang4, Roger Schibli1,3, Linjing Mu1,3, Ronny R. Buechel1, Philipp A. Kaufmann1, Simon M. Ametamey3, Catherine Gebhard1,2*, Achi Haider1,2,4*
1Department of Nuclear Medicine, University Hospital Zurich, CH-8091 Zurich, Switzerland
2Center for Molecular Cardiology, University of Zurich, CH-8952 Schlieren, Switzerland
3Institute of Pharmaceutical Sciences, ETH Zurich, CH-8093 Zurich, Switzerland
4Division of Nuclear Medicine and Molecular Imaging, Massachusetts General Hospital & Department of Radiology, Harvard Medical School, Boston, MA, 02114, United States.
Achi Haider, PhD
Institution
Picture/Logo
Optional
Copyright American Society of Nuclear Cardiology

## Slide 2
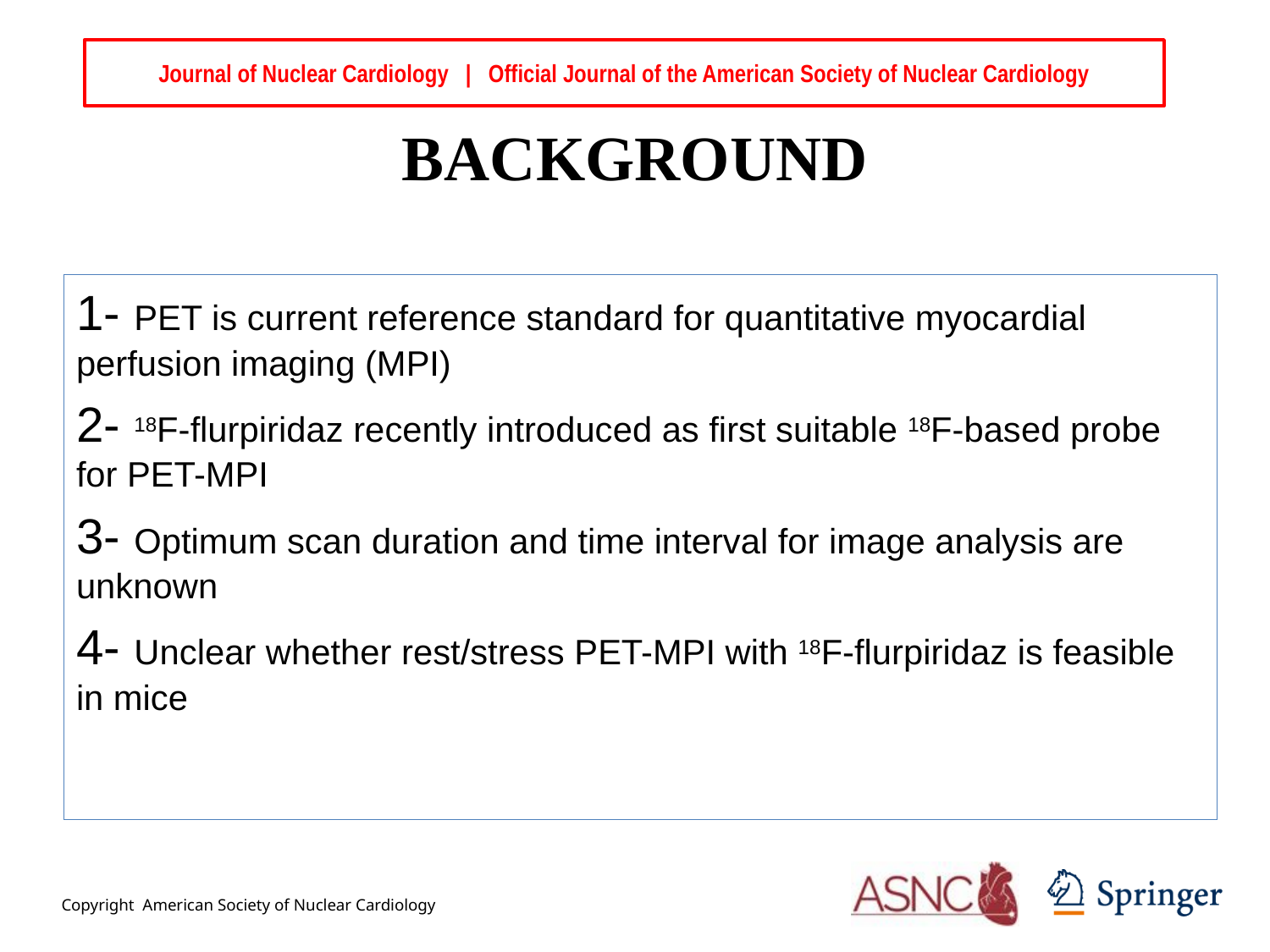

Journal of Nuclear Cardiology | Official Journal of the American Society of Nuclear Cardiology
# BACKGROUND
1- PET is current reference standard for quantitative myocardial perfusion imaging (MPI)
2- 18F-flurpiridaz recently introduced as first suitable 18F-based probe for PET-MPI
3- Optimum scan duration and time interval for image analysis are unknown
4- Unclear whether rest/stress PET-MPI with 18F-flurpiridaz is feasible in mice
Copyright American Society of Nuclear Cardiology

## Slide 3
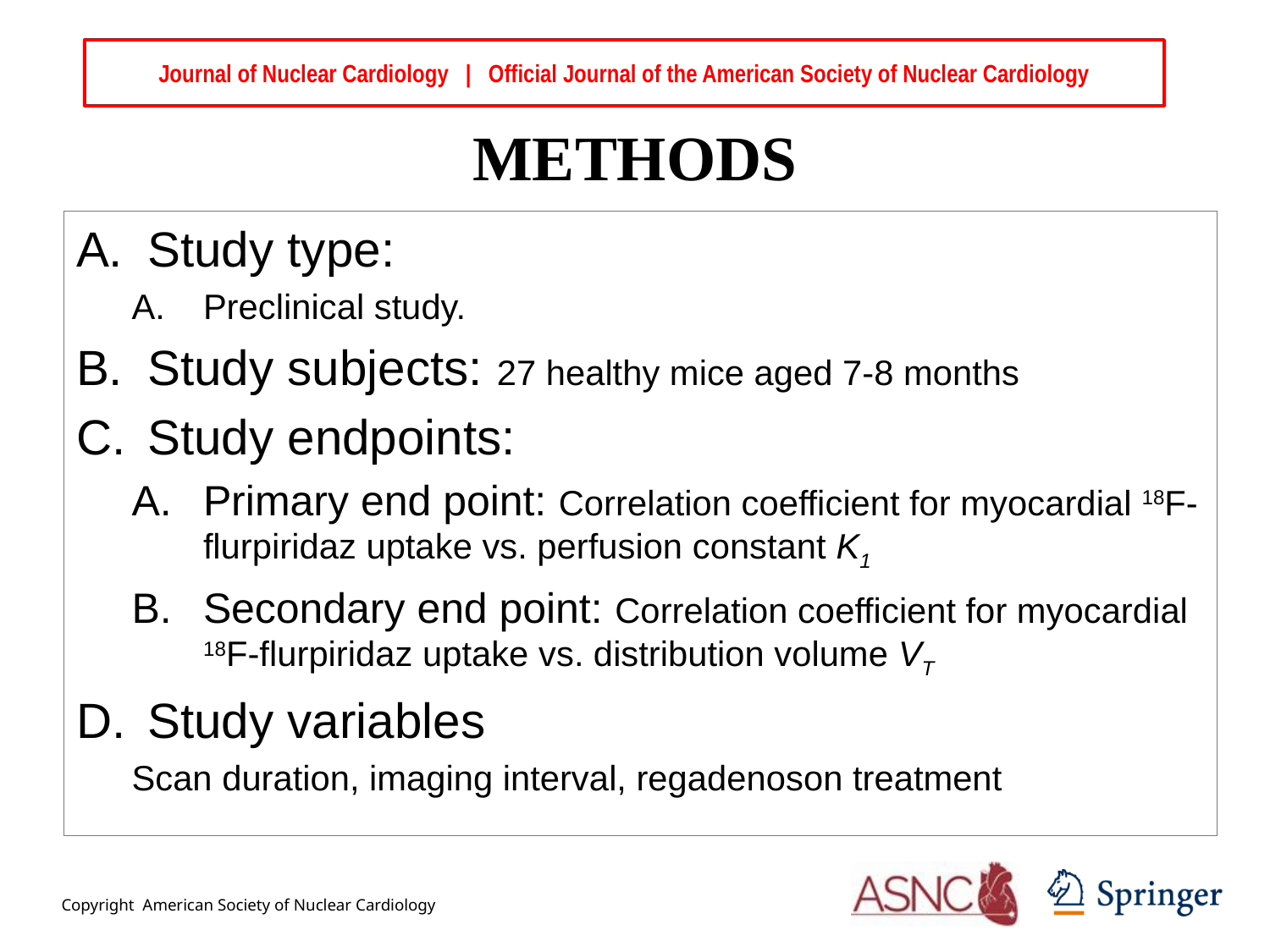

Journal of Nuclear Cardiology | Official Journal of the American Society of Nuclear Cardiology
# METHODS
Study type:
Preclinical study.
Study subjects: 27 healthy mice aged 7-8 months
Study endpoints:
Primary end point: Correlation coefficient for myocardial 18F-flurpiridaz uptake vs. perfusion constant K1
Secondary end point: Correlation coefficient for myocardial 18F-flurpiridaz uptake vs. distribution volume VT
Study variables
Scan duration, imaging interval, regadenoson treatment
Copyright American Society of Nuclear Cardiology

## Slide 4
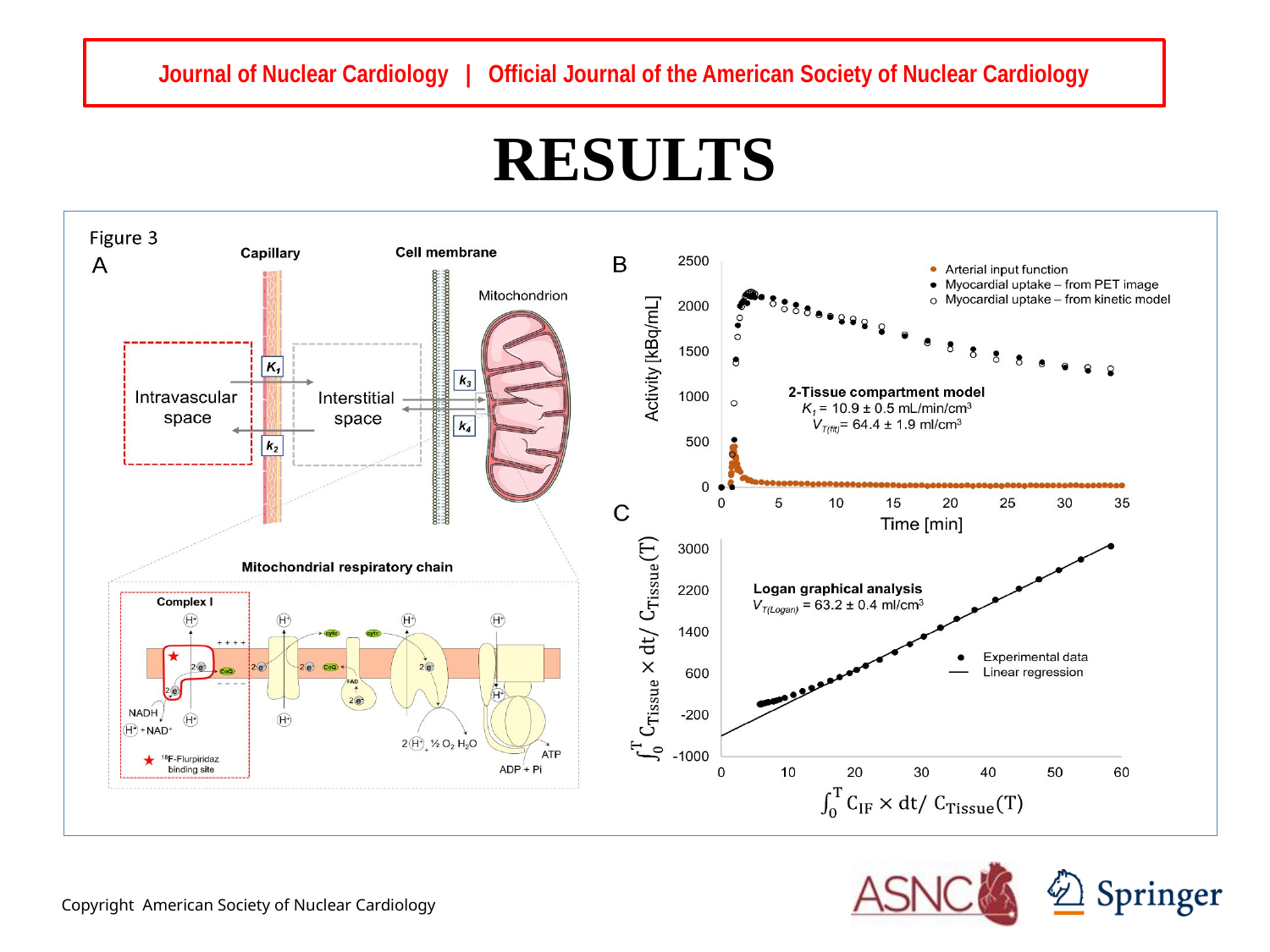

Journal of Nuclear Cardiology | Official Journal of the American Society of Nuclear Cardiology
# RESULTS
Insert a key table or a key figure
If figure, insert legend
Copyright American Society of Nuclear Cardiology

## Slide 5
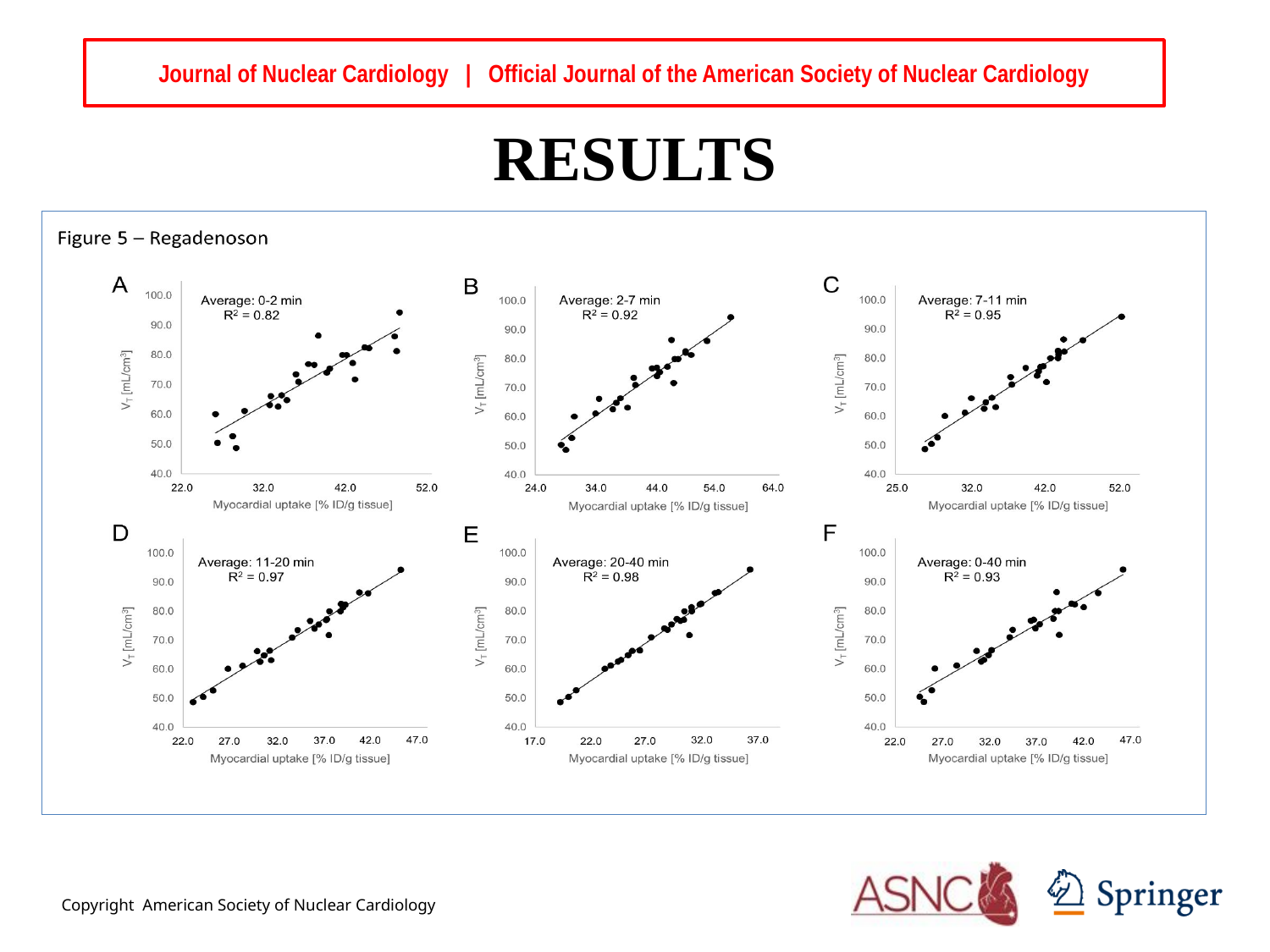

Journal of Nuclear Cardiology | Official Journal of the American Society of Nuclear Cardiology
# RESULTS
Copyright American Society of Nuclear Cardiology

## Slide 6
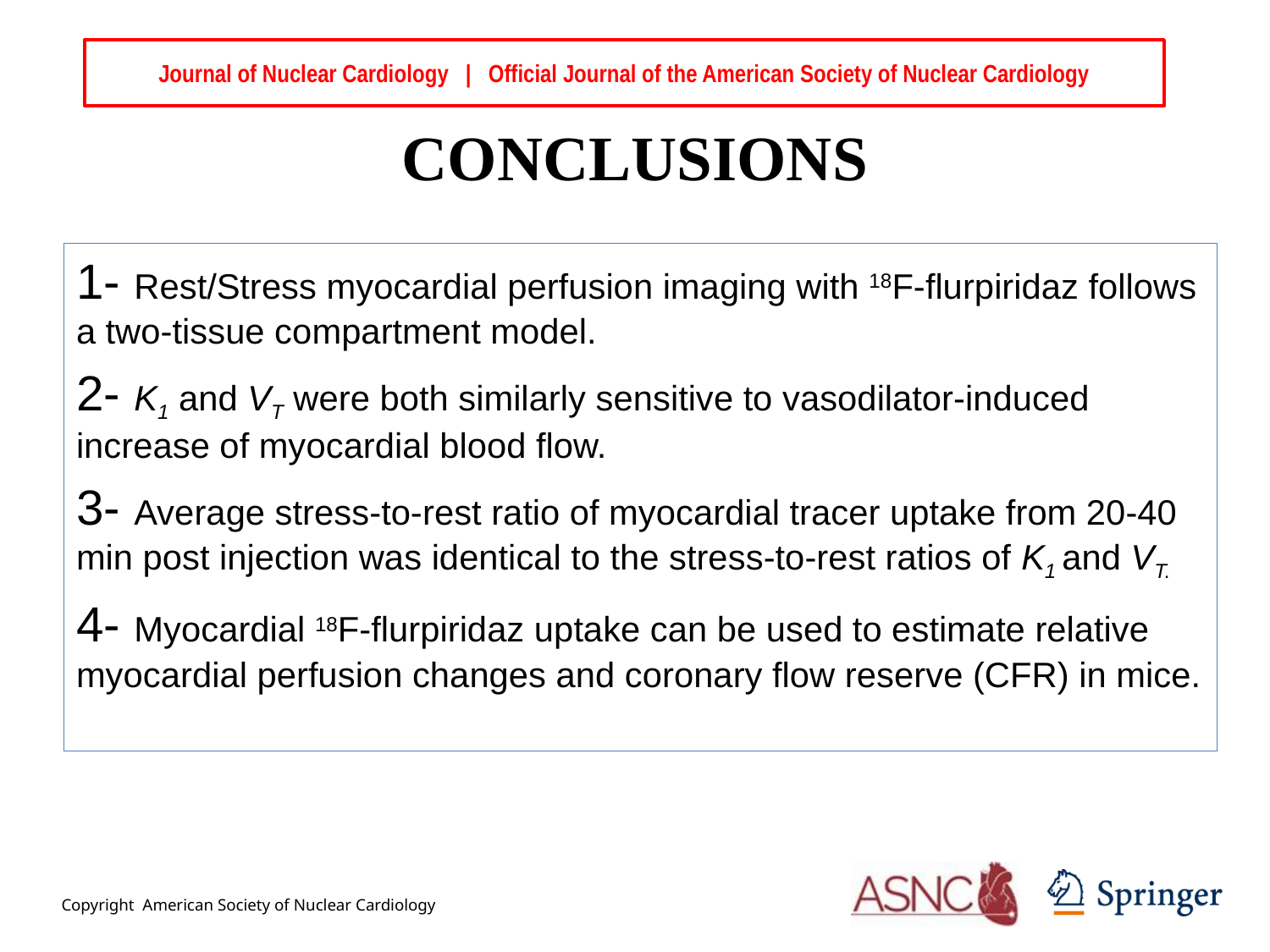

Journal of Nuclear Cardiology | Official Journal of the American Society of Nuclear Cardiology
# CONCLUSIONS
1- Rest/Stress myocardial perfusion imaging with 18F-flurpiridaz follows a two-tissue compartment model.
2- K1 and VT were both similarly sensitive to vasodilator-induced increase of myocardial blood flow.
3- Average stress-to-rest ratio of myocardial tracer uptake from 20-40 min post injection was identical to the stress-to-rest ratios of K1 and VT.
4- Myocardial 18F-flurpiridaz uptake can be used to estimate relative myocardial perfusion changes and coronary flow reserve (CFR) in mice.
Copyright American Society of Nuclear Cardiology
